# Delayed Presentation of an Intraorbital Metallic Foreign Body: A Case Report and Surgical Intervention

**DOI:** 10.7759/cureus.79701

**Published:** 2025-02-26

**Authors:** Cristiana Germano, Federica Fossataro, Vincenzo Abbate, Stefania Troise, Giovanni Dell'Aversana Orabona, Giovanni Salzano, Simona Barone, Adriana Iuliano, Diego Strianese, Paola Bonavolontà

**Affiliations:** 1 Department of Neurosciences, Reproductive and Odontostomatological Sciences, University of Naples Federico II, Naples, ITA; 2 Department of Neurosciences, Reproductive Sciences and Dentistry, University of Naples Federico II, Naples, ITA

**Keywords:** chronic orbital inflammation, diplopia, intraorbital foreign body, ocular trauma, orbital surgery

## Abstract

Ocular trauma remains a significant cause of vision loss, often resulting from penetrating injuries caused by foreign bodies such as glass, metal, wood, and other materials. Despite advancements in ocular surgery, retained intraorbital foreign bodies may lead to delayed complications if not promptly identified and managed. We present the case of a 59-year-old male who reported severe right eye pain and diplopia seven years following a work-related facial injury. Initially asymptomatic, a computed tomography (CT) scan later identified a 30-mm metallic foreign body lodged in the medial orbit. Surgical removal via anterior orbitotomy successfully alleviated symptoms, leading to the restoration of normal visual function and improvement of the patient's visual acuity to 10/10. This case highlights the importance of comprehensive evaluation in patients with a history of ocular trauma, even in the absence of immediate symptoms. While retained intraorbital foreign bodies may remain inert for extended periods, they can eventually cause chronic inflammation, pain, and other complications. CT imaging remains crucial for detecting metallic foreign bodies, and timely surgical intervention is essential to prevent further complications and preserve vision. Additionally, the case underscores the importance of preventive safety measures in the workplace to reduce the risk of such injuries.

## Introduction

Despite significant advances in traumatology, eye injuries resulting from ocular trauma remain a common cause of blindness and reduced vision [1]. These injuries can occur in various settings but are most prevalent among working individuals, particularly males [2]. Penetrating eye injuries often result from agents such as glass, thorns, wood, and sharp objects. These injuries can range from minor corneal abrasions to more severe lesions that may result in the rupture of the eye globe [2,3]. The prompt evaluation and appropriate management of ocular trauma are critical to preserving vision and preventing long-term complications [1].

Several factors are correlated with the final visual outcome in patients with eye injuries, and several neoplastic, vascular, infectious/inflammatory, and traumatic diseases can affect the orbit [4,5]. These include visual acuity, the presence of an afferent pupillary defect, the type of injury, the location and extent of the penetrating injury, the type of lens damage, the severity of vitreous hemorrhage, and the presence and composition of intraocular foreign bodies [3]. The aim of this study is to describe a rare case of an intraorbital foreign body that remained undetected for seven years following facial trauma. This case underscores the importance of comprehensive evaluation and timely intervention in cases of ocular trauma to ensure the best possible outcome for the patient.

## Case presentation

**Figure 1:**
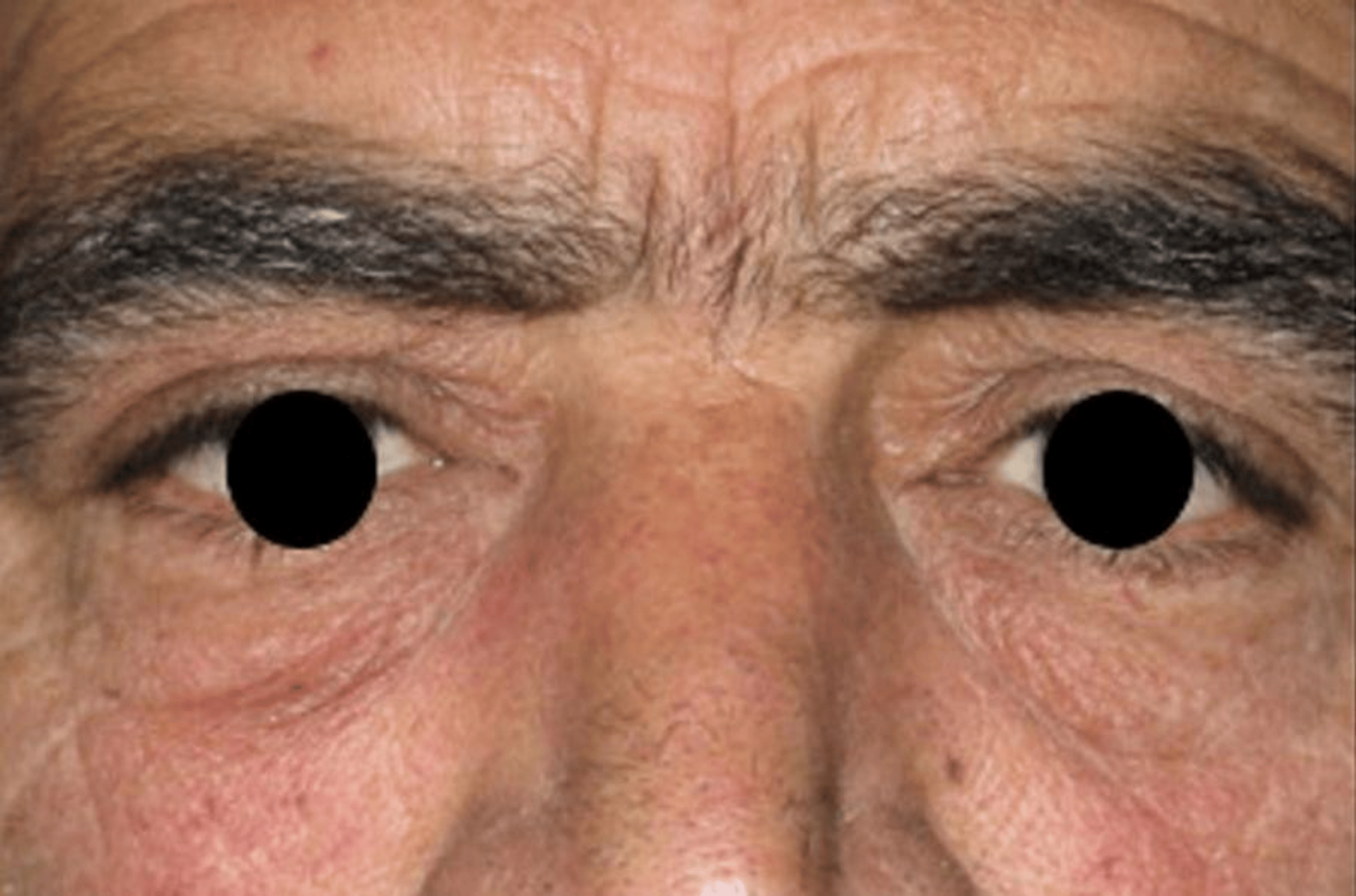
External appearance of the patient’s orbits Despite the presence of a metallic foreign body within the right orbit, the external appearance is paradoxically normal. The image shows no visible signs of trauma or abnormality. The patient provided informed consent for the use of this image, and the pupils have been obscured to maintain anonymity.

A 59-year-old man presented to the Orbital Unit of the University of Naples Federico II with severe right eye pain that had begun one month prior (Figure 1).

The patient had a history of a work-related facial injury seven years earlier, during which an intraorbital foreign body was missed and then left unremoved. Remarkably, the patient had been asymptomatic for the next seven years and had no complaints about his left eye. However, in the month preceding his visit, he experienced increasing ocular pain and began using painkillers to manage the discomfort. 

On ophthalmologic examination, the right eye's visual acuity was 9/10 on the Snellen visual acuity chart. Direct and consensual pupillary reflexes were present, and intraocular pressure was measured at 15 mmHg (normal values: 10-21 mmHg). A flashlight examination revealed no facial abnormalities, and the anterior and posterior segments of the right eye appeared normal, with no visible signs of the previous trauma. However, the patient reported diplopia in all gaze directions, which raised concerns about the underlying cause.

**Figure 2:**
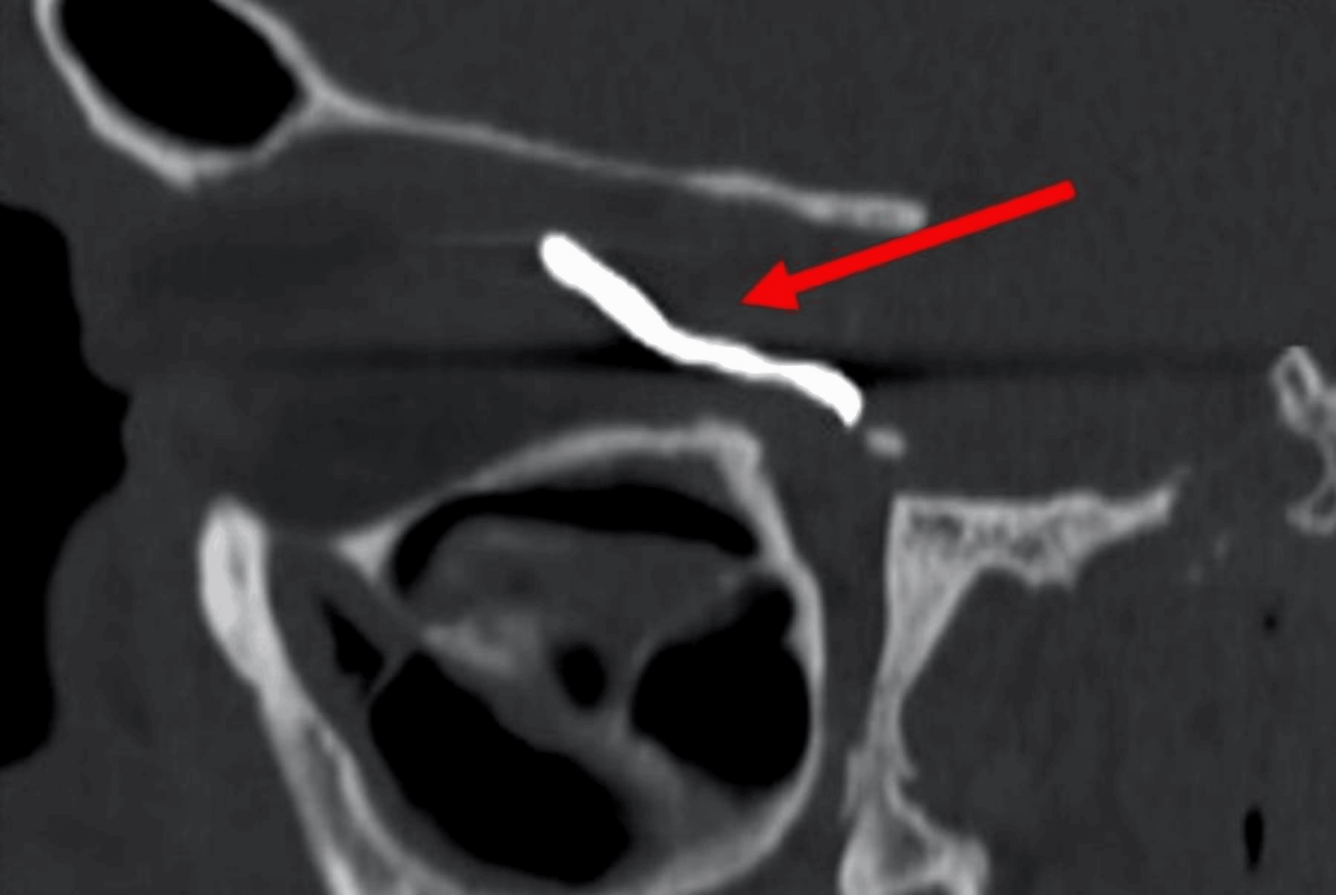
Preoperative computed tomography (CT) scan of the patient's right orbit The red arrow indicates a metallic foreign body located within the medial orbit. The foreign body appears as a hyperdense structure, consistent with the patient's clinical presentation of persistent pain and diplopia. This image emphasizes the importance of thorough imaging in cases of suspected retained intraorbital foreign bodies.

Given the patient's symptoms and history, a pre-surgical computed tomography (CT) scan of the orbit was performed. The scan revealed a high-density shadow in the medial orbit, corresponding to a metallic foreign body, with no associated bone injury (Figure 2). 

After discussing the findings and treatment options with the patient, it was decided that surgical removal of the foreign body was necessary.

**Figure 3:**
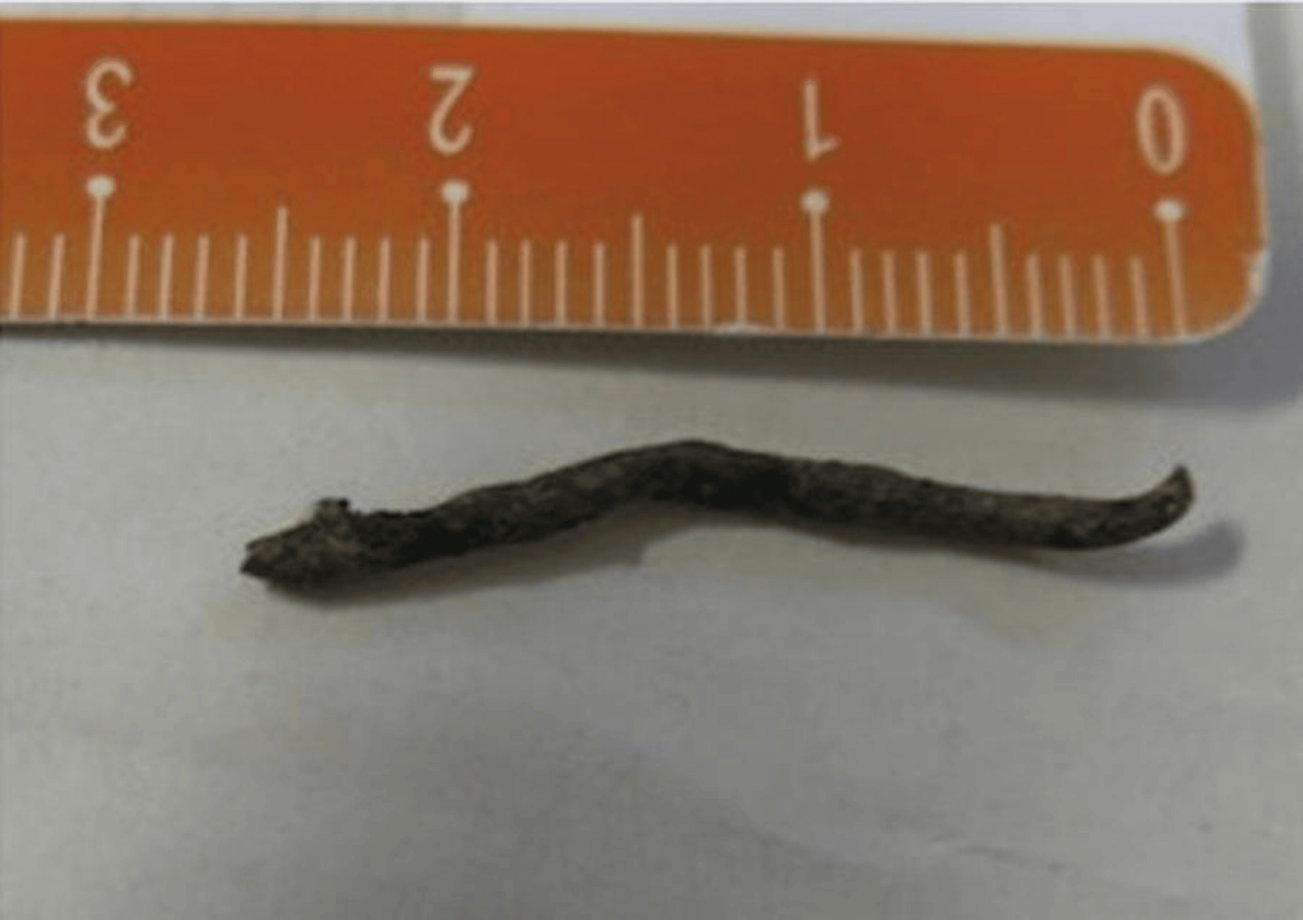
The foreign body after surgical extraction

The patient underwent anterior orbitotomy under general anesthesia, during which a metallic foreign body measuring 30 mm in length was successfully removed from the medial wall of the orbit (Figure 3). The capsule surrounding the foreign body was piecemeal removed without damaging the extrinsic ocular musculature and the ocular globe, which were not involved by the foreign body. There was no inflammatory tissue around it. Therefore, the capsule was removed, and the orbit anatomy was restored.

The procedure was uneventful, and the patient tolerated it well. Postoperatively, the patient's visual acuity in the right eye improved to 10/10, and he experienced complete resolution of pain and diplopia, with normal extraocular movements restored. The patient was initially managed with artificial tears and low-dose corticosteroids, which were progressively discontinued until the home discharge, four days after the procedure.

The patient's rapid recovery and the resolution of symptoms following surgery highlight the importance of timely intervention in cases of retained intraorbital foreign bodies. The patient was clinically and radiologically followed for seven years without neurological/visual symptoms. This case also emphasizes the need for thorough evaluation in patients with a history of ocular trauma, even if they are asymptomatic for an extended period.

## Discussion

Ocular injuries can be classified into blunt or penetrating, depending on the causative factor. Blunt injuries typically result from a forceful impact that does not penetrate the eye, while penetrating injuries involve the intrusion of a foreign object into the ocular tissues [1]. Penetrating injuries are often more severe and can lead to significant complications, particularly if a foreign body is retained within the orbit [2].

Orbital foreign bodies can be metallic or non-metallic and may include materials such as tree branches, chopsticks, wood, or metal fragments [3]. These foreign bodies can be classified based on their location within the eye or their adnexal structures, including intra-global, extra-global, intramural, or adnexal foreign bodies [3]. The initial evaluation and identification of penetrating ocular injuries or foreign bodies are crucial in minimizing further complications. Management and prognosis depend on several factors, including the composition and location of the foreign body, as well as the presence of secondary infections or other complications [3].

In this case, the patient remained asymptomatic for seven years because the metallic foreign body was inert and well-tolerated by the surrounding tissues. However, the patient eventually developed pain and diplopia, which were likely due to chronic orbital inflammation and the formation of a surrounding granuloma. When left untreated, this case demonstrates that even small foreign bodies can cause delayed complications, including chronic orbital inflammation, osteomyelitis, thrombotic vasculitis, and sepsis. Additionally, complications such as orbital hematoma, cellulitis, diplopia, proptosis, abscess formation, and blindness can occur.

CT scans are commonly used in the evaluation of orbital trauma, particularly for identifying metallic foreign bodies, which typically exhibit high density on imaging [1,6]. In contrast, magnetic resonance imaging (MRI) is generally avoided in cases involving metallic foreign bodies due to the risk of exacerbating tissue damage and causing further complications [7]. Once a foreign body is identified, the treatment typically involves surgical removal, especially if the patient is symptomatic or if there is a risk of further complications [2]. Several endoscopic and open microsurgical approaches, more or less invasive, can be selected to address the orbital cavity [8]. The surgical approach depends on the location of the foreign body within the orbit [9]. In some cases, such as when the foreign body is small and located in a challenging area like the orbital apex, surgical removal may not be necessary if the patient remains asymptomatic, as the surgery itself carries risks of complications, including orbital bleeding and optic nerve injury [6,8].

In our case, the decision to surgically remove the foreign body was based on the patient's symptoms and the potential for further complications. In this case, the successful outcome underscores the importance of a thorough evaluation and a carefully considered treatment plan. The patient's recovery and resolution of symptoms highlight the benefits of timely surgical intervention in cases of retained intraorbital foreign bodies.

Orbital trauma remains a common cause of visual impairment, particularly among working individuals who are at higher risk of injury [9]. A detailed medical history, prompt ocular examination, thorough pre-surgical evaluation, and timely intervention are essential for achieving the best possible outcomes in cases of ocular trauma. This case report highlights the importance of recognizing the potential for delayed complications in patients with retained intraorbital foreign bodies, even when they remain asymptomatic for an extended period.

## Conclusions

In this case, the successful surgical removal of the metallic foreign body resulted in the complete resolution of symptoms and the restoration of normal visual function. This outcome underscores the importance of early diagnosis and intervention to prevent early or sometimes long-term complications that can occur many years later. Additionally, the case emphasizes the need for safety precautions in the workplace to reduce the risk of ocular injuries and prevent future incidents. Education and awareness about the importance of protective eyewear and other safety measures are crucial in minimizing such injuries.
